# Context is everything: extrinsic signalling and gain-of-function p53 mutants

**DOI:** 10.1038/s41420-020-0251-x

**Published:** 2020-03-23

**Authors:** Ivano Amelio, Gerry Melino

**Affiliations:** 1 Department of Experimental Medicine, TOR, University of Rome ’’Tor Vergata”, 00133 Rome, Italy; 2 School of Life Sciences, University of Nottingham, Nottingham, UK; 3 Toxicology Unit, University of Cambridge, Department of Pathology, Tennis Court Road, Cambridge, CB2 1QP UK

**Keywords:** Cancer genetics, Tumour-suppressor proteins

## Abstract

The *TP53* genomic locus is a target of mutational events in at least half of cancers. Despite several decades of study, a full consensus on the relevance of the acquisition of p53 gain-of-function missense mutants has not been reached. Depending on cancer type, type of mutations and other unidentified factors, the relevance for tumour development and progression of the oncogenic signalling directed by p53 mutants might significantly vary, leading to inconsistent observations that have fuelled a long and fierce debate in the field. Here, we discuss how interaction with the microenvironment and stressors might dictate the gain-of-function effects exerted by individual mutants. We report evidence from the most recent literature in support of the context dependency of p53 mutant biology. This perspective article aims to raise a discussion in the field on the relevance that context might have on p53 gain-of-function mutants, assessing whether this should generally be considered a cell non-autonomous process.

## Facts

- Mutant p53 GOF effects have been shown to vary in different settings, potentially depending on cancer types, experimental models and conditions.
- Reciprocal interaction between microenvironment and mutant p53 GOF effects exists.
- Similarly to wt p53, mutant p53 proteins can contribute to transduction of extrinsic signalling.

## Questions

- What is the effective contribution of mutant p53 GOF effects to the cancer progression?
- How relevant is the microenvironmental context in determining mutant p53 GOF effects?
- Can microenvironmental factors be targeted to abolish mutant p53 GOF pro-oncogenic functions?

### p53 protects cells from insults: why do cancers want to lose this benefit?

**Fig. 1:**
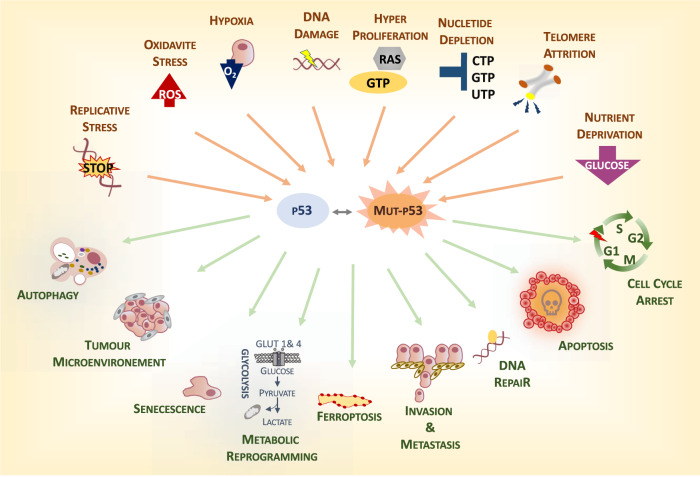
p53 is a stress response protein. p53 represents a molecular hub in the interaction between extrinsic factors and the cellular biological response. In response to stress, functional p53 exerts a protective role by promoting different mechanisms, which include apoptosis, DNA repair and senescence, as well as rewiring of cellular metabolism, autophagy etc. In presence of p53 mutants, this physiological response can be altered leading to aberrant cellular processes supporting tumorigenesis. This might underlie the GOF effect of p53 mutants.

p53 is a stress response protein^1–5^. Originally identified as a major executor of the response to DNA damage, with a more recent revision, p53 is considered a molecular hub for the interactions between stressors (reactive oxygen radicals [ROS], nutrient deprivation, hypoxia, telomere erosion, etc.) and cellular biological responses^5–12^ (Fig. 1). This view leads us to postulate that functional p53 prevents cancer via multiple mechanisms; however, this could also be more simplistically interpreted as the general role of p53 in protecting the cell in response to multiple types of stressors, which in most cases result in the prevention of tumorigenesis. Occasionally, however, efforts in defending the cell from potentially damaging factors might cause p53 to protect cancer cells. Thus, specific circumstances have emerged in which functional p53 appears to help cancer cells cope with stressors, and its contribution is beneficial for tumour progression^13–16^. The ultimate goal of p53 function of protecting the cell results in a tumour-suppressive function in the context of normal cells and represents a prototypical example of the complexity and context dependency of tumour-suppressive mechanisms.

p53 is, however, inactivated in at least 50% of human cancers, indicating that cancer cells receive a selective advantage in losing p53 function^17^. A hallmark of p53-mutated cancer cells is the loss of control of genomic integrity, which long ago led to the popular definition of p53 as the “Guardian of the Genome”^18^. Multiple mechanisms have been proposed, including the capability of p53 to transcriptionally control the DNA repair machinery^19^, to promote the death of highly damaged cells^20–22^ and to prevent retrotransposons and mobile elements from hopping across the genome^23,24^. The high genomic instability of p53 mutant tumours is not essential for the initiation of cancer; however, it strongly facilitates progression of the disease, providing the plasticity required to adapt to the constantly changing conditions within the tumour ecosystem. Thus, the genetic plasticity associated with p53 mutations is definitely an advantage for cancer cells.

An additional critical aspect of the mutations in p53 is the frequently observed protein products. Eighty percent of p53 mutations are missense, leading to generation of neomorphic proteins^25,26^, the function of which has been associated with deregulation of a wide range of physiological cellular signalling processes^14^ and interacting partners, including its family members, p63^27–29^ and p73^30–34^. These mechanisms are thought to support tumorigenesis, leading to the postulation of the gain-of-function (GOF) theory in p53 mutation^26^. The shift of p53 from the wild-type status to the mutant protein therefore appears to turn p53 into an oncogene. This basic, consistent and generally accepted consideration has, however, not always found solid support in the experimental data. Clear GOF phenotypes have been shown for many hot-spot mutations, such as p53 R175H and R273H. Introduction of p53 R175H and R273H into p53-null cells promotes growth in in vitro soft-agar assays and in injected nude mice. In contrast, genetically engineered mouse models carrying mouse homologous mutations (p53 R172H and R270H) did not show any alteration in survival compared with p53-null mice.

Puzzling results have also been shown in myeloid malignancies. GOF p53 R172H was seen to accelerate complex-karyotype acute myeloid leukaemia in mouse models by promoting cell fate plasticity through the pluripotency factor FOXH1^35,36^. However, a complementary approach based on functional and transcriptional analyses of CRISPR-Cas9-generated isogenic human leukaemia cell lines suggested a selective advantage associated with a dominant negative effect of p53 mutants but no evidence of any GOF. This study also reported no evidence of GOF effects on the clinical outcome of patients with myeloid malignancies^37^. Whether this is different from solid tumours, where a pro-invasion and pro-metastatic programme might benefit from mutant p53 GOF, remains to be determined. However, formal evidence for the existence of p53 mutant GOF effects could be found in the dependency displayed by cancers on sustained expression of p53 mutant proteins^38^. Ablation of mutant p53 in allotransplanted, xenotransplanted and autochthonous mouse cancer models impairs tumour growth, promoting cell death and tumour regression or stagnation^39–42^. While these results allow pharmacological targeting of the mechanisms leading to p53 mutant stabilisation as an anticancer approach, at the basic molecular level, they formally demonstrate that expression of p53 mutant proteins is not equal to the loss of the functional wild-type version. However, a general consensus has not yet been achieved on the relevance of the contribution of p53 GOF effects to tumorigenesis.

Recent evidence has indicated that GOF mutants functionally interact with microenvironmental factors^41,43^. This is reflected in an influence of extrinsic signalling on p53 mutant behaviour and on the ability of the p53 mutant to modulate the response to extrinsic factors. In light of the central role of functional (wt) p53 in the response to cellular stress, a fundamental conserved implication of the mutant proteins in the interaction with microenvironment is not surprising (Fig. 1). The question is therefore how much context matters in the oncogenic properties of the GOF mutant and whether GOF effects are dynamic biological processes that depend on the specific microenvironmental conditions.

### Do p53 mutants need to be activated?

p53 mutants are generally considered highly stable proteins; however, this assumption is true only in the context of tumour tissues^44,45^. Genetically modified mice carrying p53 missense mutations indeed have very low levels of protein in untransformed tissues^46^, and the stability of p53 mutants is heterogeneous within the tumour mass in humans and mice. The mutant protein conserves the wild-type intrinsically unstable nature associated with a very tight regulation of proteasomal degradation, which is mediated by MDM2 and CHIP^47^.

**Fig. 2:**
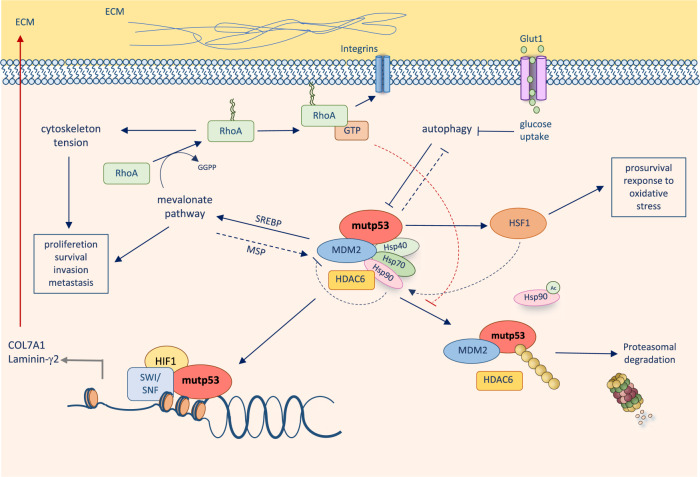
p53 mutants interaction with extracellular matrix. ECM stiffness induces HDAC6/ Hsp90-dependent stabilisation of mutant p53 with a molecular mechanism involving mevalonate pathway-dependent Rho-A geranylgeranylation. Mutant p53 in turn sustains mevalonate pathway activity providing a forward loop, which facilitates its protein stabilisation. In addition to that, in hypoxic microenvironment, mutant p53 promotes expression of ECM components, which then can trigger stabilisation of mutant p53.

Stress-responsive systems of molecular chaperones, involving Hsp70, Hsp90 and Hsp40/DNAJA1, counteract p53 mutant degradation and modulates its conformational plasticity and stability^48,49^. The master transcription factor heat-shock factor-1 (HSF1) transcriptionally promotes the expression of Hsps, which in turn interact with p53 mutants, inhibit the E3 ubiquitin ligase activity of MDM2 and CHIP and induce the stability of p53 mutant proteins. The mechanisms leading to the stabilisation of p53 mutants are therefore influenced by extrinsic stressors able to trigger the activation of HSF1. Similarly, mechanical stimuli from the tumour tissues have also been shown to result in p53 mutant stabilisation. Rho-A is a sensor of extracellular matrix (ECM) stiffness. In response to stiff a ECM, Rho-A activation and actin-dependent mechanotransduction induce activation of Hsp90 via the mechanosensitive HDAC6 deacetylase, thus leading to stabilisation of mutant proteins^50^. Similar to HSF1, Rho-A is also frequently hyperactive in tumour tissues. Notably, Rho-A is regulated by mutant p53 activity itself. Activation of Rho-A is influenced by GEF-H1 and RhoGDI, which are influenced by GOF p53 mutants, feeding a forward loop that results in further stabilisation of the p53 mutant. Disruption of this loop with zoledronic acid (ZA) and geranylgeranyl transferase inhibitors (GGTIs), which inhibit the geranylgeranylation of Rho-A required for its activation on the plasma membrane, impairs p53 mutant stability^26,43,51^ (Fig. 2). Cancer fibrosis has been associated with a high expression level of the p53 mutant and represents a possible explanation for its heterogeneous expression within the tumour tissue^52^.

The functional interaction between Rho-A and mutant p53 pulls the influence of cellular metabolism into the equation of p53 mutant stability. Geranylgeranylation of Rho-A requires activation of the mevalonate pathway, as this supplies the substrates for the synthesis of the isoprenoid geranylgeranyl pyrophosphate^50^. This led to the suggestion of repurposing statins as a potential strategy to reduce p53 mutant stability^48^. The selective pharmacological effects of statins on p53 mutant breast cancer cells have, however, also found alternative explanations. p53 mutant GOF effects have been reported in the context of activation of the mevalonate pathway via deregulation of SREBP2 transcriptional effects. Hence, p53 mutant breast cancer cells have been suggested to be selectively vulnerable to statins, as p53 mutant-mediated promotion of the mevalonate pathway facilitates invasiveness and disruption of mammary architecture in 3D culture^53^. Once again, a feed-forward loop between the pathway regulating p53 mutant stability and the reciprocal influence of the p53 mutant on the same pathway emerges, pointing out the complexity of the interaction between cell-autonomous and non-cell-autonomous factors at the basis of these molecular networks.

These data indicate that, before being important in defining p53 mutant function, the context is crucial for determining whether the mutant protein is expressed or not. Similar to the wild-type functional protein, p53 mutants respond to extrinsic signalling, which leads to stabilisation of the protein. A convergence of mechanisms conserved from the wild-type protein and newly acquired by the mutant protein leads to “activation” of the p53 GOF mutant. Whether GOF effects are executed might therefore depend on the extent of activation received by the p53 mutant protein.

### p53 mutants instruct the microenvironment

The context dependency of oncogenic and oncosuppressive signalling has now clearly emerged. The simplistic cell-autonomous model of the oncogenic effects mediated by hyperactive mitogenic mutated proto-oncogenes has been widely revised with the increasing complexity of the oncogenic effects of mutant proteins and the contribution of extrinsic signalling. Cellular fate appears to be dictated by the integration of the genetic landscape and microenvironmental cues.

In addition to the impact on protein stability, a stringent relationship exists between p53 mutants and the microenvironment; p53 mutants have emerged as a significant contributor to shaping the microenvironmental conditions.

Central factors of the tumour microenvironment, such as the ECM composition, appear to be influenced by mutant p53 GOF. A cooperation between p53 mutants and hypoxia-inducible factor-1 (HIF-1) determines the expression of fundamental components of the basal lamina, such as laminin-γ2 and collagen type VIIa1, in hypoxic tumours^41^. The result of the cooperation between the p53 mutant and HIF-1 is the generation of a pro-tumorigenic microenvironment that facilitates the invasion of lung cancer cells and the growth of xenotransplanted tumours^41,54,55^. Remarkably, the trigger of this p53 mutant GOF requires hypoxia to initiate the HIF-1-mediated response; thus, again, a reciprocal interaction between extrinsic and intrinsic factors participates in the cascade of events (Fig. 2).

GOF p53 mutants can also facilitate a pro-tumorigenic microenvironment by promoting the secretion of IL-8 and GRO-α by cancer cells. Similar to the p53 mutant/HIF-1 molecular model, p53 mutants appear to hijack the E2F2 transcriptional factor. The p53 mutant/E2F2 complex controls the promoter of inhibitor of DNA binding 4 (ID4), which binds and stabilise mRNAs of IL-8 and GRO-α. Conditioned media from p53 mutant cells promote the in vitro proliferation of endothelial cells, and p53 mutants favour angiogenesis in vivo in xenotransplanted models^56^. Consistently, vessel density correlates with the expression of p53 missense mutations in human breast cancers^57^.

N-glycosylation of cellular surface proteins, such as receptors and integrins, is also altered by p53 status. Ectonucleoside triphosphate diphosphohydrolase 5 (ENTPD5) is a mutant p53 target gene involved in promoting the folding of N-glycosylated membrane proteins in the endoplasmic reticulum^58^. Cooperation between mutant p53 and Sp1 facilitates transcriptional control of ENTPD5, and this appears to mediate p53 mutant-dependent growth, architectural tissue remodelling, migration, invasion and lung colonisation as assessed in tail vein-implantation mouse models^58^.

Evidence exists that p53 mutants can influence the microenvironment. Simplification tends to indicate how “mutants” direct the composition of the extracellular milieu; however, it is reasonable to speculate that different mutations will have different impacts on this process. However, the large variety of mutations makes the resolution of this issue very challenging (Fig. 1).

### Conclusion: p53 mutants: one gene, many proteins in many contexts

**Fig. 3:**
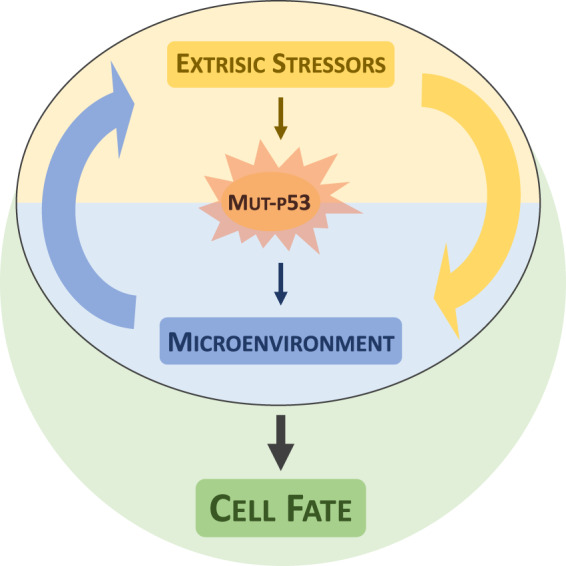
Integration between extrinsic stressors and p53 status determines cellular fate. Mutant p53 function is influenced by extrinsic stressors and in turn can shape the tumour microenvironments. Integration of extinct stressors and mutant p53-depedent signalling can determine cellular fate. Thus, mutant p53 GOF might be a context dependent process.

In this perspective, we suggest that p53 GOF is a non-cell-autonomous phenomenon, which would make this gene very peculiar if compared with other oncogenes/oncosuppressors, such as KRas^59,60^, Rb^61,62^ or Bcl-2^63–67^. The p53 mutant can dictate instructions to the microenvironment, which in turn are influenced by additional extrinsic factors that direct signals on the cancer cells that are differentially integrated based on the p53 mutational status (Fig. 3). Thus, in the regulatory circle established between cancer cells and the microenvironment, p53 mutants represent a molecular hub determining the outcome.

It is remarkable that a significant fraction of work on p53 GOF is performed in in vitro systems or in xenotransplanted models. Compelling evidence indicates that plastic dishes provide altered extracellular matrix interactions to cancer cells, influencing the stability of p53 mutants and consequentially providing artificial responses^51^. The limited ability of in vitro and xenotransplanted models to recapitulate the natural cancer microenvironment might represent a particular concern in the interpretation of the data on p53 GOF. The addiction that cancer cells develop to p53 mutations is a specific relevant aspect in this context. While strong support for the existence and relevance of p53 GOF comes from evidence that in vivo tumours are dependent on p53 expression, no evidence exists of the reproducibility of this effect in vitro. This questions not only the work done to dissect the mechanisms underlying p53 GOF but also the utility of in vitro models for developing therapeutic approaches to target p53 mutant stability.

It appears optimistic to envisage general major mechanisms responsible for p53 GOF, but it is possibly also very optimistic to envisage the existence of p53 mutant of similar mechanisms in different contexts. This raises complexity in the study of p53 GOF, but even more, it presents challenges in identifying strategies to target this gene with general approaches in cancer patients. While a massive effort in drug development against p53 mutant tumours has been invested in the two past decades, the outcome is very soon expected to be delivered. This should also help in understanding how feasible the approach is and direct efforts for future investments^68^.

## References

[1] Laptenko O, Prives C (2017). p53: master of life, death, and the epigenome. Genes Dev..

[2] Labuschagne CF, Zani F, Vousden KH (2018). Control of metabolism by p53—cancer and beyond. Biochim. Biophys. Acta Rev. Cancer.

[3] Aubrey BJ, Kelly GL, Janic A, Herold MJ, Strasser A (2018). How does p53 induce apoptosis and how does this relate to p53-mediated tumour suppression?. Cell Death Differ..

[4] Engeland K (2018). Cell cycle arrest through indirect transcriptional repression by p53: I have a DREAM. Cell Death Differ..

[5] Kaiser AM, Attardi LD (2018). Deconstructing networks of p53-mediated tumor suppression in vivo. Cell Death Differ..

[6] Mello SS, Attardi LD (2018). Deciphering p53 signaling in tumor suppression. Curr. Opin. Cell Biol..

[7] Pitolli C (2019). p53-mediated tumor suppression: DNA-damage response and alternative mechanisms. Cancers.

[8] Sullivan KD, Galbraith MD, Andrysik Z, Espinosa JM (2018). Mechanisms of transcriptional regulation by p53. Cell Death Differ..

[9] Chen Y, Liu K, Shi Y, Shao C (2018). The tango of ROS and p53 in tissue stem cells. Cell Death Differ..

[10] Xu R (2018). Tumor suppressor p53 links ceramide metabolism to DNA damage response through alkaline ceramidase 2. Cell Death Differ..

[11] Sankunny M, Eng C (2018). KLLN-mediated DNA damage-induced apoptosis is associated with regulation of p53 phosphorylation and acetylation in breast cancer cells. Cell Death Discov..

[12] Amelio I, Melino G (2015). The p53 family and the hypoxia-inducible factors (HIFs): determinants of cancer progression. Trends Biochem. Sci..

[13] Tajan M (2018). A role for p53 in the adaptation to glutamine starvation through the expression of SLC1A3. Cell Metab..

[14] Maddocks OD (2013). Serine starvation induces stress and p53-dependent metabolic remodelling in cancer cells. Nature.

[15] Humpton TJ, Vousden KH (2016). Regulation of cellular metabolism and hypoxia by p53. Cold Spring Harb. Perspect. Med..

[16] Amelio I, Cutruzzola F, Antonov A, Agostini M, Melino G (2014). Serine and glycine metabolism in cancer. Trends Biochem. Sci..

[17] Kastenhuber ER, Lowe SW (2017). Putting p53 in context. Cell.

[18] Lane DP (1992). Cancer. p53, guardian of the genome. Nature.

[19] Janic A (2018). DNA repair processes are critical mediators of p53-dependent tumor suppression. Nat. Med..

[20] Nakano K, Vousden KH (2001). PUMA, a novel proapoptotic gene, is induced by p53. Mol. Cell.

[21] Yakovlev AG (2004). BOK and NOXA are essential mediators of p53-dependent apoptosis. J. Biol. Chem..

[22] Galluzzi L (2018). Molecular mechanisms of cell death: recommendations of the Nomenclature Committee on Cell Death 2018. Cell Death Differ..

[23] Wylie A (2016). p53 genes function to restrain mobile elements. Genes Dev..

[24] Leonova KI (2013). p53 cooperates with DNA methylation and a suicidal interferon response to maintain epigenetic silencing of repeats and noncoding RNAs. Proc. Natl Acad. Sci. USA.

[25] Pitolli, C. et al. Do mutations turn p53 into an oncogene? *Int. J. Mol. Sci.* **20**, 10.3390/ijms20246241 (2019). 10.3390/ijms20246241 PMC6940991 31835684

[26] Mantovani F, Collavin L, Del Sal G (2019). Mutant p53 as a guardian of the cancer cell. Cell Death Differ..

[27] Adorno M (2009). A mutant-p53/Smad complex opposes p63 to empower TGFbeta-induced metastasis. Cell.

[28] Muller PA (2009). Mutant p53 drives invasion by promoting integrin recycling. Cell.

[29] Celardo I (2013). Caspase-1 is a novel target of p63 in tumor suppression. Cell Death Dis..

[30] Amelio I (2014). TAp73 promotes anabolism. Oncotarget.

[31] Nemajerova A (2018). Non-oncogenic roles of TAp73: from multiciliogenesis to metabolism. Cell Death Differ..

[32] Lena AM (2010). Skn-1a/Oct-11 and DeltaNp63alpha exert antagonizing effects on human keratin expression. Biochem. Biophys. Res. Commun..

[33] Weissmueller S (2014). Mutant p53 drives pancreatic cancer metastasis through cell-autonomous PDGF receptor beta signaling. Cell.

[34] Amelio I (2015). TAp73 opposes tumor angiogenesis by promoting hypoxia-inducible factor 1alpha degradation. Proc. Natl Acad. Sci. USA.

[35] Loizou E (2019). A gain-of-function p53-mutant oncogene promotes cell fate plasticity and myeloid leukemia through the pluripotency factor FOXH1. Cancer Discov..

[36] Amelio I (2019). How mutant p53 empowers Foxh1 fostering leukaemogenesis?. Cell Death Discov..

[37] Boettcher S (2019). A dominant-negative effect drives selection of TP53 missense mutations in myeloid malignancies. Science.

[38] Kim MP, Lozano G (2018). Mutant p53 partners in crime. Cell Death Differ..

[39] Alexandrova EM (2015). Improving survival by exploiting tumour dependence on stabilized mutant p53 for treatment. Nature.

[40] Schulz-Heddergott R (2018). Therapeutic ablation of gain-of-function mutant p53 in colorectal cancer inhibits Stat3-mediated tumor growth and invasion. Cancer Cell.

[41] Amelio I (2018). p53 mutants cooperate with HIF-1 in transcriptional regulation of extracellular matrix components to promote tumor progression. Proc. Natl Acad. Sci. USA.

[42] Furth N, Aylon Y, Oren M (2018). p53 shades of Hippo. Cell Death Differ..

[43] Ingallina E (2018). Mechanical cues control mutant p53 stability through a mevalonate-RhoA axis. Nat. Cell Biol..

[44] Lang GA (2004). Gain of function of a p53 hot spot mutation in a mouse model of Li-Fraumeni syndrome. Cell.

[45] Terzian T (2008). The inherent instability of mutant p53 is alleviated by Mdm2 or p16INK4a loss. Genes Dev..

[46] Donehower LA, Lozano G (2009). 20 years studying p53 functions in genetically engineered mice. Nat. Rev. Cancer.

[47] Wu D, Prives C (2018). Relevance of the p53-MDM2 axis to aging. Cell Death Differ..

[48] Parrales A (2016). DNAJA1 controls the fate of misfolded mutant p53 through the mevalonate pathway. Nat. Cell Biol..

[49] Boysen M, Kityk R, Mayer MP (2019). Hsp70- and Hsp90-mediated regulation of the conformation of p53 DNA binding domain and p53 cancer variants. Mol. Cell.

[50] Zhang FL, Casey PJ (1996). Protein prenylation: molecular mechanisms and functional consequences. Annu Rev. Biochem..

[51] Sorrentino G, Mantovani F, Del Sal G (2018). The stiff RhoAd from mevalonate to mutant p53. Cell Death Differ..

[52] Koga T (2001). Heterogeneous distribution of P53 immunoreactivity in human lung adenocarcinoma correlates with MDM2 protein expression, rather than with P53 gene mutation. Int J. Cancer.

[53] Freed-Pastor WA (2012). Mutant p53 disrupts mammary tissue architecture via the mevalonate pathway. Cell.

[54] Petrova V, Annicchiarico-Petruzzelli M, Melino G, Amelio I (2018). The hypoxic tumour microenvironment. Oncogenesis.

[55] Petrova V (2015). TAp73 transcriptionally represses BNIP3 expression. Cell Cycle.

[56] Fontemaggi G (2009). The execution of the transcriptional axis mutant p53, E2F1 and ID4 promotes tumor neo-angiogenesis. Nat. Struct. Mol. Biol..

[57] Linderholm BK (2001). The expression of vascular endothelial growth factor correlates with mutant p53 and poor prognosis in human breast cancer. Cancer Res..

[58] Vogiatzi F (2016). Mutant p53 promotes tumor progression and metastasis by the endoplasmic reticulum UDPase ENTPD5. Proc. Natl Acad. Sci. USA.

[59] Wu HZ, Xiao JQ, Xiao SS, Cheng Y (2019). KRAS: a promising therapeutic target for cancer treatment. Curr. Top. Med. Chem..

[60] Lanfredini S, Thapa A, O'Neill E (2019). RAS in pancreatic cancer. Biochem. Soc. Trans..

[61] Goel S, DeCristo MJ, McAllister SS, Zhao JJ (2018). CDK4/6 inhibition in cancer: beyond cell cycle arrest. Trends Cell Biol..

[62] Singh U, Malik MA, Goswami S, Shukla S, Kaur J (2016). Epigenetic regulation of human retinoblastoma. Tumour Biol..

[63] Adams JM, Cory S (2018). The BCL-2 arbiters of apoptosis and their growing role as cancer targets. Cell Death Differ..

[64] Strasser A, Vaux DL (2018). Viewing BCL2 and cell death control from an evolutionary perspective. Cell Death Differ..

[65] Kale J, Osterlund EJ, Andrews DW (2018). BCL-2 family proteins: changing partners in the dance towards death. Cell Death Differ..

[66] Adams CM, Clark-Garvey S, Porcu P, Eischen CM (2018). Targeting the Bcl-2 family in B cell lymphoma. Front Oncol..

[67] Afreen S, Weiss JM, Strahm B, Erlacher M (2018). Concise review: cheating death for a better transplant. Stem Cells.

[68] Levine AJ (2018). Reviewing the future of the P53 field. Cell Death Differ..

